# Supplementary material for: Phylogeography and Population Variation in Prunus discoidea (Prunus subg. Cerasus) in China
Source: Plants (Basel). 2024 Sep 9;13(17):2535. doi: 10.3390/plants13172535 (PMC11396878; doi:10.3390/plants13172535)
Supplement: Supplementary file 1 [file plants-13-02535-s001.zip › plants-3176728-supplementary.pdf]

Table S1. Results of neutral test in population division of *Prunus discoidea*

Statistics	BMQ	BYS	DMS	ZJS	HS	LS	YTS	SMS	ZJG	LKY	THC	LCS	YZH
	**chloroplast DNA fragments**												
Tajima'sD	2.53769	0.00000	1.70339	2.13208	2.35898	0.00000	1.50518	1.30634	-0.77806	2.13321	0.17954	2.15610	1.63481
P-value	0.99600	1.00000	0.97100	0.98100	0.99300	1.00000	0.96400	0.92000	0.24200	0.97200	0.61200	0.99300	0.94000
Fu'sFs	6.64627	0.00000	1.19443	-0.79797	2.96257	0.00000	1.40457	1.67306	1.75666	3.67982	1.212661	4.54620	-1.25618
P-value	0.99300	N.A.	0.76800	0.39600	0.92200	N.A.	0.69400	0.85000	0.76600	0.96900	0.77300	0.97400	0.284000
	**nuclear DNA fragments**												
Tajima'sD	-0.59000	1.26352	1.29541	0.00000	1.96274	0.00000	0.00000	0.83775	2.30624	0.00000	0.00000	2.76247	2.54383
P-value	0.28300	0.87000	0.90600	1.00000	0.97700	1.00000	1.00000	0.83500	0.98900	1.00000	1.00000	0.99500	0.99200
Fu'sFs	1.01952	2.83999	6.57871	0.00000	9.40807	0.00000	0.00000	2.17676	5.82751	0.00000	0.00000	9.95939	5.30106
P-value	0.54700	0.89000	0.99500	N.A.	1.00000	N.A.	N.A.	0.81000	0.99600	N.A.	N.A.	1.00000	0.99200

Table S2. Neutral test of geographical grouping of *Prunus discoidea*

Statistics	Central	Eastern	All
	**chloroplast DNA fragments**		
Tajima’sD	1.84703	0.49778	0.92153
P-value	0.94800	0.67800	0.77500
Fu’sFs	-1.43840	-0.06667	-3.40693
P-value	6.34100	6.53700	0.14700
	**nuclear DNA fragments**		
Tajima’sD	1.44001	2.71428	1.25492
P-value	0.90400	0.99600	0.82700
Fu’sFs	6.16471	12.46840	7.46592
P-value	0.97400	1.00000	0.98900

Table S3. Mismatch distribution analysis (AMOVA) of *Prunus discoidea*

Statistics	BMQ	BYS	DMS	ZJS	HS	LS	YTS	SMS	ZJG	LKY	THC	LCS	YZH
	**chloroplast DNA fragments**												
Demographic expansion													
SSD	0.49598	0.00000	0.021508	0.01118	0.05453	0.00000	0.02746	0.02339	0.06222	0.18083	0.43848	0.25251	0.01106
P-value	0.00000	0.00000	0.38000	0.10000	0.18000	0.00000	0.09000	0.47000	0.04000	0.08000	0.00000	0.06000	0.12000
Raggedness index	0.74800	0.00000	0.10260	0.06556	0.19370	0.00000	0.27549	0.11111	0.71293	0.53760	0.17324	0.74249	0.05294
P-value	0.91000	0.00000	0.64000	0.14000	0.23000	0.00000	0.04000	0.54000	0.59000	0.01000	1.00000	0.02000	0.43000
Spatial expansion													
SSD	0.17015	0.00000	0.01074	0.01092	0.03506	0.00000	0.02746	0.02366	0.02797	0.13371	0.02402	0.15480	0.00901
P-value	0.02000	0.00000	0.61000	0.07000	0.26000	0.00000	0.02000	0.31000	0.24000	0.01000	0.44000	0.01000	0.33000
Raggedness index	0.74800	0.00000	0.10260	0.06556	0.19370	0.00000	0.27549	0.11111	0.71293	0.53760	0.17324	0.74249	0.05294
P-value	0.19000	0.00000	0.75000	0.19000	0.52000	0.00000	0.07000	0.68000	0.70000	0.14000	0.57000	0.22000	0.58000
	**nuclear DNA fragments**												
Demographic expansion													
SSD	0.04066	0.32171	0.18613	0.00000	0.22771	0.00000	0.00000	0.32000	0.14333	0.00000	0.00000	0.48473	0.06431
P-value	0.03000	0.00000	0.06000	0.00000	0.02000	0.00000	0.00000	0.00000	0.06000	0.00000	0.00000	0.00000	0.13000
Raggedness index	0.74149	0.68043	0.52018	0.00000	0.68388	0.00000	0.00000	0.68000	0.42266	0.00000	0.00000	0.74249	0.08260
P-value	0.68000	1.00000	0.12000	0.00000	0.38000	0.00000	0.00000	0.89000	0.00000	0.00000	0.00000	0.92000	0.39000
Spatial expansion													
SSD	0.01685	0.08780	0.12645	0.00000	0.12932	0.00000	0.00000	0.08734	0.10327	0.00000	0.00000	0.18727	0.04102
P-value	0.24000	0.09000	0.06000	0.00000	0.13000	0.00000	0.00000	0.09000	0.10000	0.00000	0.00000	0.01000	0.18000
Raggedness index	0.74149	0.068043	0.52018	0.00000	0.68388	0.00000	0.00000	0.68000	0.42266	0.00000	0.00000	0.74249	0.08260
P-value	0.57000	0.33000	0.46000	0.00000	0.48000	0.00000	0.00000	0.48000	0.34000	0.00000	0.00000	0.27000	0.85000

Table S4. Mismatch distribution analysis (AMOVA) of *Prunus discoidea*

Statistics	Central	Eastern	All
		**chloroplast DNA fragments**	
Demographic expansion			
SSD	0.03241	0.00494	0.01938
P-value	0.05000	0.77000	0.18000
Raggedness index	0.08738	0.28830	0.05561
P-value	0.09000	0.93000	0.33000
Spatial expansion			
SSD	0.02578	0.00603	0.01489
P-value	0.07000	0.66000	0.38000
Raggedness index	0.08738	0.02883	0.05661
P-value	0.2000	0.92000	0.53000
		**nuclear DNA fragments**	
Demographic expansion			
SSD	0.07947	0.45001	0.32821
P-value	0.08000	0.00000	0.00000
Raggedness index	0.45344	0.56042	0.74249
P-value	0.56000	0.94000	0.92000
Spatial expansion			
SSD	0.06239	0.12664	0.18727
P-value	0.25000	0.06000	0.01000
Raggedness index	0.44618	0.56042	0.74249
P-value	0.61000	0.43000	0.27000

Table S5. Statistical parameters of demographic and spatial expansion in *P. discoidea*

Statistics	All_Seqs	Mean	Standard Deviation
Demographic expansion			
Tau	2.94727	2.94727	0
Tau qt 2.5%	0.01563	0.01563	0
Tau qt 5%	0.88867	0.88867	0
Tau qt 95%	5.08594	5.08594	0
Tau qt 97.5%	5.70508	5.70508	0
Theta0	0.00176	0.00176	0
Theta0 qt 2.5%	0	0	0
Theta0 qt 5%	0	0	0
Theta0 qt 95%	0.40781	0.40781	0
Theta0 qt 97.5%	1.31484	1.31484	0
Theta1	5.33203	5.33203	0
Theta1 qt 2.5%	2.77832	2.77832	0
Theta1 qt 5%	3.76709	3.76709	0
Theta1 qt 95%	99999	99999	0
Theta1 qt 97.5%	99999	99999	0
SSD	0.01938	0.01938	0
Model (SSD) p-value	0.13	0.13	0
Raggedness index	0.05661	0.05661	0
Raggedness p-value	0.26	0.26	0
Spatial expansion			
Tau	2.59588	2.59588	0
Tau qt 2.5%	0.49936	0.49936	0
Tau qt 5%	0.74633	0.74633	0

Tau qt 95%	3.72741	3.72741	0
Tau qt 97.5%	4.36392	4.36392	0
Theta	0.00443	0.00443	0
Theta qt 2.5%	0.00072	0.00072	0
Theta qt 5%	0.00072	0.00072	0
Theta qt 95%	1.00528	1.00528	0
Theta qt 97.5%	1.27914	1.27914	0
M	5.92814	5.92814	0
M qt 2.5%	0.30245	0.30245	0
M qt 5%	0.65089	0.65089	0
M qt 95%	99999	99999	0
M qt 97.5%	99999	99999	0
SSD	0.01489	0.01489	0
Model (SSD) p-value	0.4	0.4	0
Raggedness index	0.05661	0.05661	0
Raggedness p-value	0.64	0.64	0

Note: Tau represents the expansion time, Theta represents the effective population size, and M represents the migration rate.

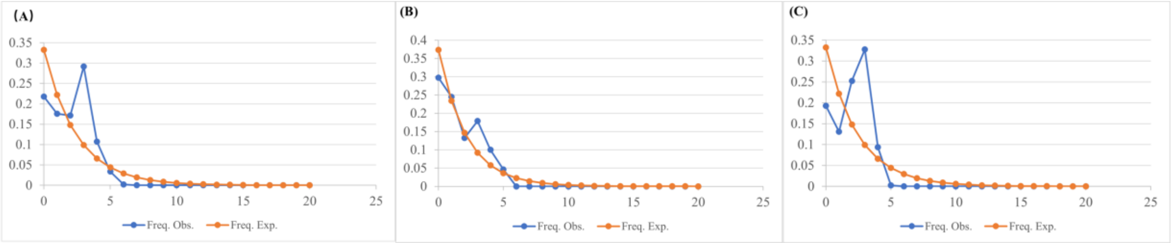

Figure S1. Mismatch analysis of different geographical combinations of *P. discoidea* groups based on the cpDNA (*rpoB*, *rps16*, *trnD–E*) sequences.

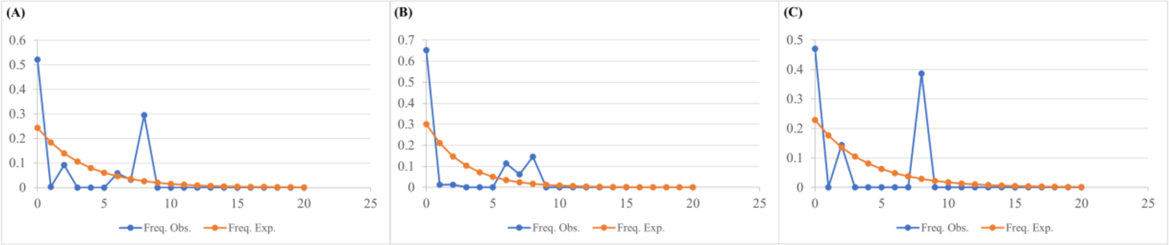

Figure S2. Mismatch analysis of different geographical combinations of P. discoidea groups based on the nrDNA (ITS) sequences.
